# Surface engineered porous silicon for stable, high performance electrochemical supercapacitors

**DOI:** 10.1038/srep03020

**Published:** 2013-10-22

**Authors:** Landon Oakes, Andrew Westover, Jeremy W. Mares, Shahana Chatterjee, William R. Erwin, Rizia Bardhan, Sharon M. Weiss, Cary L. Pint

**Affiliations:** 1 Department of Mechanical Engineering, Vanderbilt University, Nashville, TN 37235, USA; 2 Department of Electrical Engineering and Computer Science, Vanderbilt University, Nashville, TN 37235, USA; 3 Department of Chemical and Biomolecular Engineering, Vanderbilt University, Nashville, TN 37235, USA; 4 Interdisciplinary Materials Science Program, Vanderbilt University, Nashville, TN 37235

## Abstract

Silicon materials remain unused for supercapacitors due to extreme reactivity of silicon with electrolytes. However, doped silicon materials boast a low mass density, excellent conductivity, a controllably etched nanoporous structure, and combined earth abundance and technological presence appealing to diverse energy storage frameworks. Here, we demonstrate a universal route to transform porous silicon (P-Si) into stable electrodes for electrochemical devices through growth of an ultra-thin, conformal graphene coating on the P-Si surface. This graphene coating simultaneously passivates surface charge traps and provides an ideal electrode-electrolyte electrochemical interface. This leads to 10–40X improvement in energy density, and a 2X wider electrochemical window compared to identically-structured unpassivated P-Si. This work demonstrates a technique generalizable to mesoporous and nanoporous materials that decouples the engineering of electrode structure and electrochemical surface stability to engineer performance in electrochemical environments. Specifically, we demonstrate P-Si as a promising new platform for grid-scale and integrated electrochemical energy storage.

The prospect for viable solutions to future energy storage challenges requires the active materials for energy storage to be produced from low-cost sources for grid-scale applications, or from materials compatible with processes and systems for consumer-level electronics applications^1^,^2^. Silicon is the 2^nd^ most abundant element on the planet and has been a material with revolutionary impact on the electronics and solar industries. These industries have driven production of silicon raw materials to a range of $2–$30 per kilogram ranging from metallurgical grade to electronic grade, respectively. However, doped silicon suffers both from surface traps that inhibit conductivity^3^ and the immense reactivity of surface-bound silicon atoms with electrolytes that inhibits electrochemical stability^4^,^5^. This reactivity has favored the wide use of silicon as anode materials in metal-ion batteries^6^,^7^, where charge is stored through intercalation reactions, but has inhibited producing silicon-based materials for stable double-layer charge storage. Until now, there have been only a few investigations of silicon materials in electrochemical environments^8^,^9^,^10^,^11^, noting specific capacitances in device configurations orders of magnitude lower (5 mF/g) than carbon materials for on-chip micro-supercapacitors^9^,^10^, and a strong dependence of the equivalent series resistance (ESR) on the surface characteristics of the silicon^11^. Our results demonstrate over two orders of magnitude improvement in device performance compared to previously published reports.

On the other hand, there is a rich field focused around nanostructured carbon materials for supercapacitor applications as well as the development of new architectures of meso- and nanoporous materials. Materials such as graphene and carbon nanotubes serve as platforms for excellent specific device performance due to an inherent electrochemical stability and good electrical conductivity^12^,^13^,^14^,^15^, but face limitations due to the assembly of porous, high-surface area templates that can maintain electrical interconnection and be controllably produced for mechanistic optimization. In such devices, volumetric performance is often overlooked due to inhomogeneity in sample thickness despite the industrial importance of volumetric storage characteristics when assessing performance for mobile technology and transportation applications^16^. This underlines a structural advantage of controllable porous materials, such as porous silicon, since the electrochemical etch process or fabrication process that forms the active material structure dictates the volumetric energy storage properties and enables this metric to be easily assessed and controlled^17^,^18^,^19^,^20^. In fact, there exist a variety of types of porous materials, such as metal-organic frameworks (MOFs)^21^,^22^, mesoporous materials^23^,^24^, and electrochemically etched porous semiconductors^25^ where fine control over surface area, porosity, and pore morphology is achieved utilizing controlled techniques, such as directing molecular building blocks into porous structures for MOFs, for example. In this manner, a key focus of the work presented here is to emphasize a route to utilize the structural control offered by such porous materials, which is challenging to achieve in conventional carbon nanomaterials, and modify the surface stability of these materials for activation as controlled templates viable for electrochemical energy storage – a concept we demonstrate here for porous silicon templates. The chip-based etch process for silicon also yields a structure where the active silicon material forms a robust mechanical interface with the doped silicon collector material, making this ideal for integration into applications without the formation of a distinct interface between the active energy storage material and the device. Given the abundance of silicon photovoltaics, sensors, and electronics, energy storage integration onto excess silicon material in these devices without the need for complex packaging is a practical route toward efficient, integrated energy storage systems.

## Results

**Figure 1:**
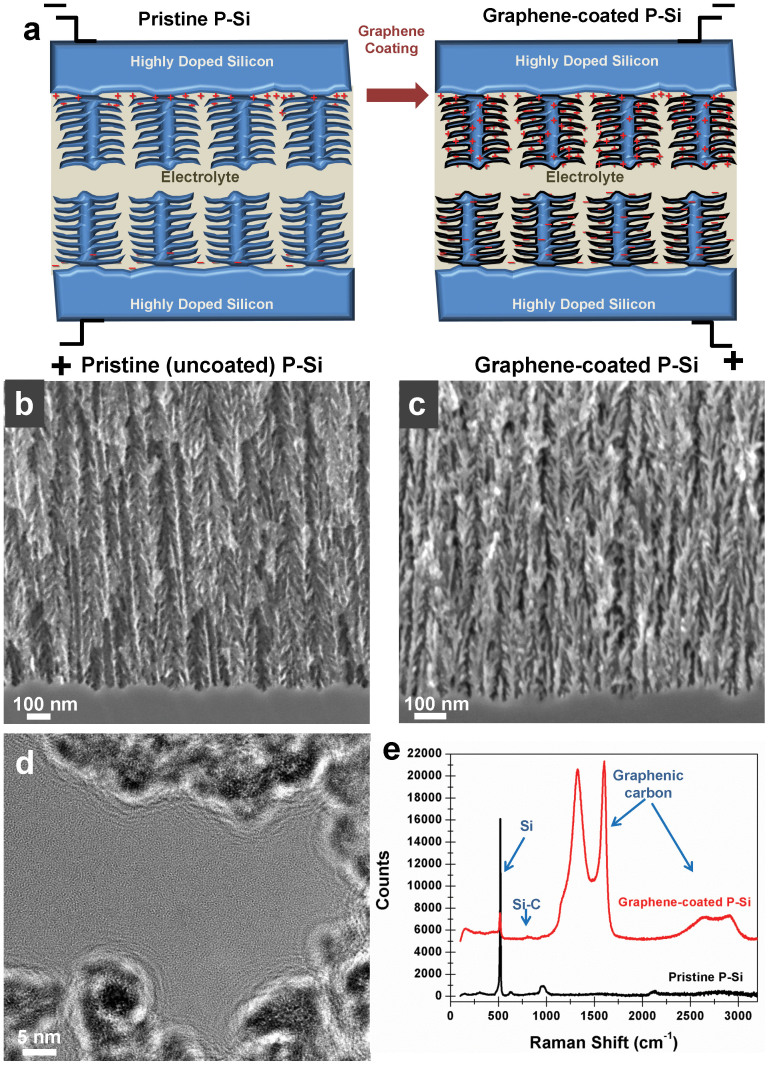
Graphene coating on porous silicon. (a). Scheme of the effect of coating P-Si on the capacitive charge storage properties. SEM cross-sectional images of porous silicon showing the interface between the etched porous silicon and the silicon wafer for the case of (b). uncoated, pristine porous silicon and (c). graphene coated porous silicon. (d). Cross-sectional TEM image of graphene-coated porous silicon structures (scale bar = 5 nm). (e). Raman spectroscopy taken at 785 nm showing pristine P-Si and graphene-coated P-Si, with the carbon, Si, and Si-C peaks labeled.

**Figure 2:**
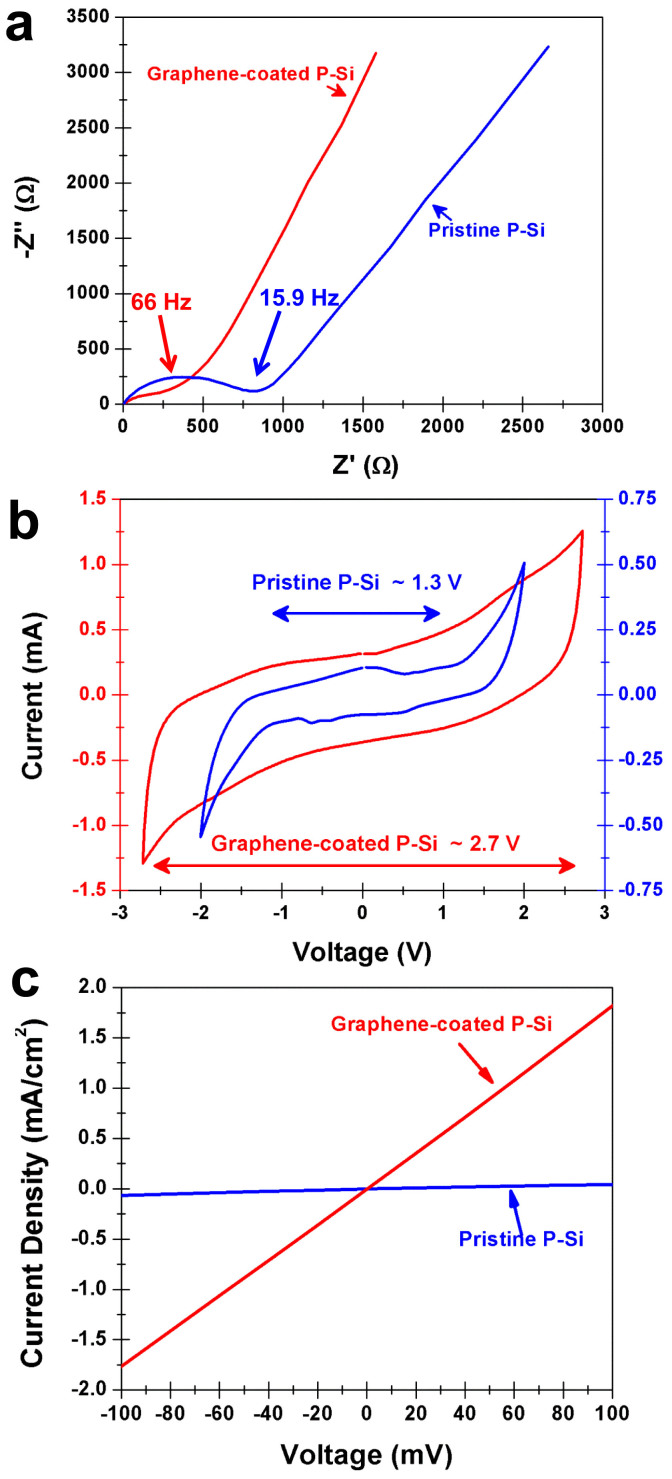
Electrical and electrochemical effects of coating graphene on porous silicon. (a). Nyquist plot for graphene-coated and pristine P-Si based on EIS sample characterization, with knee frequencies labeled in the plot. (b). Cyclic Voltammetry measurements for graphene-coated and pristine P-Si, with approximate electrochemical windows in EMIBF4 electrolyte environment labeled, and (c). through-plane electrical measurement I-V curves of graphene-coated and pristine P-Si samples emphasizing a dramatic decrease in sample resistance due to the presence of graphene.

**Figure 3:**
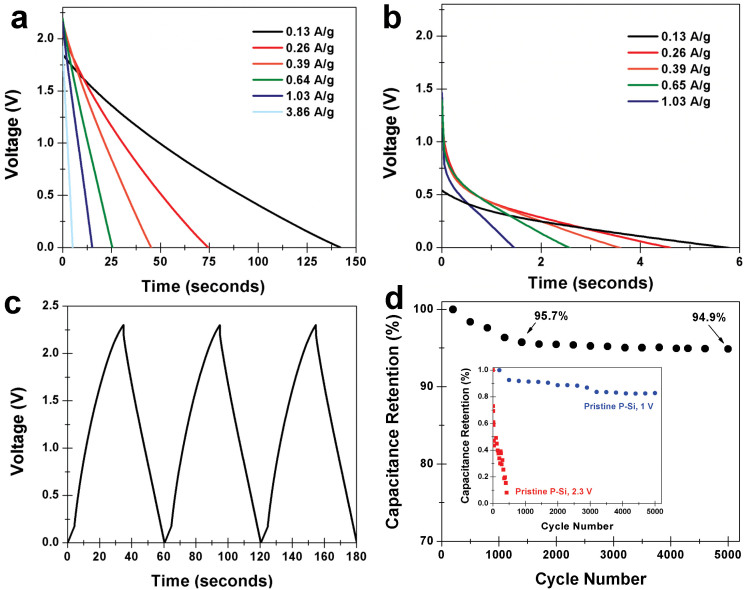
Supercapacitor charge-discharge characteristics of coated and uncoated porous silicon. (a–b) Galvanostatic discharge curves for (a). graphene-coated P-Si and (b). uncoated, pristine P-Si at different, consistent charging currents after charging to 2.3 V in EMIBF_4_ electrolyte. (c). three consecutive charge-discharge curves taken at 0.65 A/g for graphene-coated P-Si, showing the triangular charge-discharge curve. (d). Capacitance retention over 5000 cycles measured for graphene-coated P-Si, with retention % labeled at ~1500 and 5000 cycles. Inset in this is capacitance retention for pristine P-Si cycled both at 2.3 V (same as graphene-coated P-Si) and at 1 V inside the electrochemical window.

**Figure 4:**
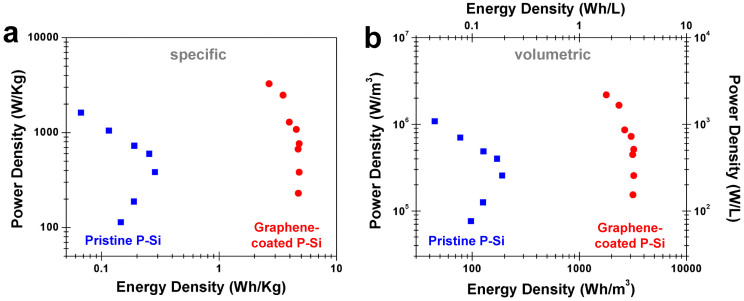
Specific and volumetric Ragone plots for coated and pristine porous silicon Ragone analysis for pristine, uncoated P-Si (blue, squares) and graphene-coated P-Si (red, circles) in the framework of both (a). specific and (b). volumetric energy storage characteristics.

A scheme of both a pristine and graphene carbon coated porous silicon (P-Si) ultracapacitor device is shown in Fig. 1a. P-Si is etched from highly doped (0.01–0.02 Ωcm) silicon wafers using a current density of 45 mA/cm^2^ in a 50% HF and ethanol solution (3:8 v/v). This etch condition was optimized to yield ~75% porosity, and samples were etched to a thickness of ~4 μm, confirmed *via* SEM imaging (Fig. S8). After etching, the P-Si samples were treated with C_2_H_2_/H_2_/Ar gas mixtures over a temperature ramp from 650°C to 850°C extending ~20 minutes. The temperature ramp was critical to both inhibit P-Si melting and to form a stable, passive coating of graphene. Results presented in Figs. 2**–**4 and in the supporting information indicate that the role of this graphene coating is to both restore conductivity to the silicon active material as well as to form a stable electrode-elecrolyte interface that is critical to achieve good energy storage characteristics. This leads to much greater capacitive charge storage for the graphene-coated devices, despite an identical porous structure, as illustrated in Fig. 1a.

Scanning electron microscope (SEM) images of P-Si before (Fig. 1b) and after (Fig. 1c) coating with graphene reveal nanoscale features giving rise to high surface areas that enable this material architecture for electrochemical supercapacitor electrodes. Graphene coating of identically etched P-Si does not alter the nanoscale architecture. Furthermore, imaging these materials using transmission electron microscopy (TEM) in Fig. 1d indicates a thin coating of uniform graphene layers that are observed between 5–10 layers thick (up to 3 nm), coated onto silicon nanostructures with an intermediate, brighter interface that we ascribe to a mixed Si-C mixed interface. Such layers of graphene are found to fill pores with diameters less than ~2–3 nm (Fig. S4). To analyze the chemical characteristics of the carbon and silicon following the gas-phase chemical treatment, we performed Raman spectroscopy (Figure 1e) of samples before and after graphene coating. Prior to coating, the most notable feature is the strong Si Raman peak near 520 cm^−1^. After coating, peaks distinctive to carbon emerge at 1325 cm^−1^ and 1602 cm^−1^. These peaks have been observed in defective graphene materials that are grown using non-catalytic growth techniques^26^, and are clearly distinguished from the Raman signature of amorphous carbon^27^. Whereas this material is henceforth denoted as graphene, it is a form of graphene-like carbon that exhibits a significant amount of sp^3^ hybridized carbon as evidenced by the D-band (~1325 cm^−1^). We expect the presence of this sp^3^ hybridized carbon to arise due to the prevalent sub-5 nm pore features that dictate the inclusion of a significant presence of sp^3^ carbons in graphene to maintain a curved architecture necessary for conformality on the Si. Additionally, the emergence of a small, broad peak is observed near 800 cm^−1^ where Raman modes of Si-C commonly appear in nanostructures^28^. Previous work focused on graphitization of P-Si using greater levels of carbon have also noted the presence of Si-C when thermal treatments exceeded ~600°C^29^. We expect that graphene growth in our case is mediated by the near-melting characteristics of P-Si (Fig. S1, S2) that generates a stable Si-C interface to catalyze growth at low temperatures (650–850°C), as opposed to greater than 1300°C on bulk SiC^30^.

In order to assess the effect of coating graphene onto P-Si, we performed both electrochemical and electrical testing (Fig. 2). Samples were prepared using 1-ethyl-3-methylimidazolium tetrafluoroborate (EMIBF_4_) ionic liquid electrolytes and vacuum infiltration into P-Si. Electrochemical impedance spectroscopy (EIS) measurements (Fig. 2a) indicate substantial improvement in the electrochemical properties of the graphene-coated P-Si samples compared to pristine P-Si. The knee frequency in the Nyquist plot corresponds to the upper limit frequency cutoff for double-layer energy storage, and the semicircle observed in the pristine P-Si sample corresponds to charge transfer processes at the silicon-EMIBF_4_ interface^31^,^32^,^33^. In accordance with equivalent circuit modeling discussed in the supporting information (Fig. S5, Table S1), the absence of a semicircle for graphene-coated P-Si corresponds to a charge-transfer resistance at the electrode-electrolyte interface that is lower than the pristine P-Si by over 30×. This indicates a device with better ionic conductivity at the electrode-electrolyte interface. The higher knee frequency (65 Hz versus 15.9 Hz) for the graphene-coated porous Si also emphasizes a stable double-layer formed over a wider range of frequencies in comparison to the pristine P-Si, and the decreased slope of the mid-frequency spike for uncoated P-Si corresponds to a ~15× lower conductivity for diffusion of ions into the porous structure, represented by the Warburg diffusion element (Table S1). This analysis emphasizes that the surface properties of a porous material play a significant role to dictate ideal electrochemical device performance.

Cyclic voltammetry (CV) measurements were also performed on identical graphene-coated P-Si and pristine P-Si at a scan rates from 25–100 mV/second, (Fig. S6) with a comparison between graphene-coated and pristine porous silicon shown at 50 mV/sec (Fig. 2b). The graphene-coated P-Si, structurally identical to the pristine P-Si, exhibits a ~2× greater electrochemical window. The electrochemical window for a supercapacitor is defined at voltages where a stable double-layer is formed at the electrode-electrolyte interface without the occurrence of Faradaic reactions. For pristine P-Si, Faradaic reactions occur above ~1.3 V, as indicated by an exponential increase in current above this voltage. CV curves for graphene-coated P-Si indicate a substantially enhanced electrochemical window to voltages near ~2.7 V. Whereas graphene supercapacitor devices have demonstrated operation up to ~4 V with EMIBF_4_ electrolytes^34^, we expect our voltage window to be limited by defective sp^3^ sites in graphene (Fig. 1e) that initiates Faradaic reactions with the electrolyte above 2.7 V. Higher voltage operation should be possible with high quality graphene coatings on the P-Si. Also evident from the CV curves is the substantial improvement in average capacitance for the coated P-Si indicating better charge storage properties. To better understand these observations, we performed through-substrate two-terminal electrical tests of both the graphene-coated and pristine P-Si samples contacting a flat stainless steel electrode. The resistance is a relative measure of the electrical conductivity through the P-Si layer in both cases since the doped silicon is highly conductive. The I-V curves obtained (Fig. 2c) indicate that the graphene-coated P-Si has ~50× lower resistance than pristine P-Si, attributable to the presence of surface traps in pristine P-Si that reverse doping effects. This correlates well to the ~50× improvement in internal electrochemical device resistance for graphene-coated porous silicon based upon equivalent circuit modeling (Table S1). Previous reports have also documented P-Si to be several orders of magnitude less conductive than doped bulk silicon^3^. We propose that the coating of P-Si with graphene passivates the P-Si surface to restore conductivity while simultaneously generating an electrode-electrolyte interface compatible with non-Faradaic charge storage. This supports the notion illustrated in the pristine P-Si panel of Fig. 1a, where ions can only be stored on conductive electrode-electrolyte interfaces, or near to the collector electrode, limiting the total capacitance.

In order to assess the device-level behavior of these P-Si supercapacitors, we performed charge-discharge measurements in a homemade test cell with two similarly sized P-Si electrodes and a Celgard membrane separator. Figures 3a and 3b show Galvanostatic discharge curves from graphene-coated P-Si (a) and pristine P-Si (b) at different charge-discharge currents after being charged to 2.3 V, which is within the measured electrochemical window for graphene coated P-Si. It is evident the discharge time scales in Fig. 3a and 3b are significantly different using identical specific charge and discharge currents. Under charging to 2.3 V, the pristine P-Si samples exhibit a large power drop that prevents discharge at voltages higher than 1 V. However, for graphene-coated P-Si devices, the linear discharge curve resembles a well-behaved supercapacitor, with discharge times over 20× greater than the pristine P-Si devices. This trend also holds true for P-Si devices cycled to 1 V, which is inside the electrochemical window for P-Si (Fig. S7). Furthermore, as shown in Fig. 3c, the shape of the charge-discharge curve for the graphene-coated devices is consistently triangular, with a Coulombic efficiency over 90%. Therefore the application of a few-nanometer coating of graphene can transform a silicon material poorly suited as a supercapacitor electrode into a well-behaved device with no structural modifications. These trends are further emphasized by repetitive device cycling results plotted in Fig. 3d. In this case, 5000 cycles were performed with a voltage of 2.3 V at a charging rate of ~1 A/g for the graphene-coated samples. Whereas the device exhibits ~4% loss of capacitance across the first 1500 cycles, the last 3500 cycles demonstrate long-term device stability due to only 0.8% capacitance change. This is in contrast to pristine porous silicon devices which exhibit over 20% capacitance degradation when cycled at 1 V for 5000 cycles, and full degradation of the device when cycling at the same voltage as the graphene-coated device (2.3 V) for less than 500 cycles.

Using data from electrochemical device testing, a Ragone plot was constructed (Figure 4) to emphasize the energy-power characteristics of these devices for both specific (Fig. 4a) and volumetric (Fig. 4b) performance. The controllable and well-defined dimensions of the P-Si enable a straight-forward determination of volumetric properties, an advantage over other double-layer capacitor materials. As shown in Figure 4, the energy-power characteristics for the pristine P-Si devices generally fall outside the range of behavior expected for a conventional supercapacitor device^1^. At low powers, (<0.5 KW/Kg), the pristine P-Si devices exhibit a decrease in energy density that we contribute to device degradation, which is not present when cycled at 1 V (Fig. S7). Above 0.5 KW/Kg, the pristine P-Si devices also show a decrease in energy density that can be attributed to only localized charge storage near conductive sites (Fig. 1a), yielding poor performance. For the graphene-coated P-Si devices, the Ragone plots are consistent with a good supercapacitor, as these devices yield up to 4.8–4.9 Wh/Kg and 3.2 KWh/m^3^ below power densities of 1 KW/Kg and 1000 KW/m^3^, respectively. These values are obtained by explicitly integrating the discharge curves, whereas up to ~7 Wh/Kg is achieved by applying a more conventional *½CV_max_*^2^ relation between the energy, capacitance and voltage. After consideration of device packaging (assuming 50% device weight), this yields ~2.5–3.5 Wh/Kg energy densities that are comparable to commercially available activated carbon-based supercapacitor devices^35^.

## Discussion

The collective picture from this work represents a comparison between two identically structured P-Si materials in the framework of electrochemical supercapacitors, with the only difference being that one is passivated with an ultra-thin coating of graphene. Our results emphasize that this passive coating of atomically-thin carbon material can transform P-Si into an electrochemical supercapacitor material with substantially improved performance relative to the uncoated material. This involves a ~3× improved knee frequency (Fig. 2a), a ~50× improved equivalent series resistance (Fig. 2c, Table S1), a ~30× improved electrolyte-electrode interface charge transfer resistance, a ~15× improved Warburg diffusion resistance (Table S1), a 2× improved electrochemical window (Fig. 2b), a 10–40× improvement in both volumetric and specific energy density (Fig. 4), and significantly enhanced long term cycling characteristics with less than 1% change in capacitance over 3500 cycles (Fig. 3b). This study therefore decouples the importance of porous electrode structure, which is identical between the two materials, and the electrochemical stability of the surface in enabling this material to exhibit ideal behavior as an electrochemical supercapacitor. Whereas both electrode pore structure and morphology and electrochemical stability are two necessary ingredients in the design of electrochemical supercapacitors, this work offers a new approach to independently design pore structure and electrochemical surface stability. This opens up new avenues for the development of new classes of electrochemically stable materials building upon advances and innovations in areas of mesoporous, microporous, and nanoporous material fabrication, where atomically-thin surface engineering can be a tool to enable functionality of such structures in environments where controlled pore structure and morphology is critical for device performance.

Overall, the specific use of silicon as an earth-abundant material capable of being transformed into a high performance electrochemical capacitor through application of a thin surface coating opens new avenues both toward grid-scale and integrated device applications. The use of processes to form on-chip, mechanically integrated devices with controllable porosity, thickness, and morphology yields promise toward integration of efficient energy storage into existent silicon-based technology platforms in diverse technologies such as solar devices, sensors, and electronics.

## Methods

### Porous silicon fabrication

Porous silicon (P-Si) etching was performed in a homemade electrochemical cell using a spiral Pt counterelectrode. Highly boron-doped (0.01–0.02 Ωcm) silicon wafers were utilized in an etch process of duration 180 seconds with a current density of 45 mA/cm^2^ in a 3:8 v/v HF (50% H_2_O by volume) and ethanol solution. The etch condition yielded ~75% porosity P-Si films confirmed by optical reflectivity measurements. Following the P-Si etch process, the samples were washed in ethanol and stored in a N_2_ glove box until gas phase carbonization.

### Graphene coating

Graphene coating was performed in a 3-zone Lindberg Blue furnace with a 4” quartz tube. P-Si samples were loaded onto a custom-fabricated sample holder and placed in the center zone of the tube furnace. The sample was then placed under vacuum, and when 2–5 mTorr was reached, 1 SLM of Ar and 200 SCCM of H_2_ was utilized to maintain atmospheric pressure in the quartz tube while the furnace was heated to 650°C. When the furnace reached 650°C, 10 SCCM of C_2_H_2_ was added to the gas mixture, and the temperature was increased to 750°C and held for 10 minutes, and increased to 850°C and held for another 10 minutes. The C_2_H_2_ was then turned off, and the P-Si was cooled in the presence of Ar and H_2_ until the sample reached a temperature of ~50°C at which it was removed from the system. After graphene coating, the samples were stored in a N_2_ glove box until devices were fabricated from these materials. Analysis of the materials before and after coating was performed with a Zeiss Merlin SEM and a Renishaw inVia MicroRaman system.

### Supercapacitor device fabrication and testing

Supercapacitor devices were fabricated by first taking P-Si materials, cutting them into ~1 cm × 1 cm pieces, and placing several drops of 1-ethyl-3-methylimidazolium tetrafluoroborate (EMIBF_4_, 99%, Sigma-Aldrich) ionic liquid onto the surface of each sample. These samples were then placed in an MTI vacuum oven for a period of 1–2 hours during which period air bubbles were observed to form while the EMIBF_4_ infiltrated into the P-Si porous structure. Following this, excess EMIBF_4_ was removed, and the samples were sandwiched into a homemade test cell separated by a Celgard 1180 separator. Samples were then tested utilizing a Metrohm Autolab Multichannel analyzer. This involved Galvanostatic charge-discharge measurements in current ranges of 0.1–5 mA, cyclic voltammetry at scan rates of 25–100 mV/second, and electrochemical impedance spectroscopy (EIS) between 1 MHz to 10 mHz. EIS measurements were performed around zero bias with a signal amplitude of 10 mV to establish device performance in an unbiased system where differences in the electrochemical stability would not influence the interpretation and equivalent circuit analysis. In order to obtain specific device performance characteristics, the active mass was calculated for the combined porous silicon and graphene material coating based upon the measured porosity, measured thickness (Figure S8), and mass of graphene coated on the porous silicon surface.

## Author Contributions

C.L.P. conceived the idea and C.L.P. and L.O. designed the experiments. L.O. and A.W. carried out the device testing and performance analysis. J.M. and S.C. etched the porous silicon materials for experiments with facilities provided by S.M.W. W.R.E. and R.B. carried out imaging studies. C.L.P. wrote the manuscript and all authors participated in discussion and reviewed the manuscript prior to submission.

## Supplementary Material

Supplementary Information Supporting Information
