# Supplementary material for: Surface engineered porous silicon for stable, high performance electrochemical supercapacitors
Source: Sci Rep. 2013 Oct 22;3:3020. doi: 10.1038/srep03020 (PMC3804850; doi:10.1038/srep03020)
Supplement: Supplementary Information — Supporting Information [file srep03020-s1.pdf]

Supplementary Information

Surface engineered porous silicon for stable, high performance electrochemical supercapacitors

Landon Oakes[1,4], Andrew Westover[1,4], Jeremy W. Mares[2], Shahana Chatterjee[1], William R. Erwin[3], Rizia Bardhan[3,4], Sharon M. Weiss[2,4], and Cary L. Pint[1,4,*]

[1]Department of Mechanical Engineering, Vanderbilt University, Nashville, TN 37235, USA

[2]Department of Electrical Engineering and Computer Science, Vanderbilt University, Nashville, TN

[3]Department of Chemical and Biomolecular Engineering, Vanderbilt University, Nashville, TN 37235, USA

[4]Interdisciplinary Materials Science Program, Vanderbilt University, Nashville, TN 37235

* To whom correspondence should be addressed. E-mail: cary.l.pint@vanderbilt.edu

I. Temperature effects in P-Si stabilization

As shown in Figure 1, porous silicon materials enable the formation of efficient supercapacitors due to the nanostructured pore morphology they exhibit. However, such small nanoscale features are known to exhibit a depressed melting temperature compared to bulk that can compete with the ability to utilize high temperature processes to stabilize the surface of the P-Si material. This was clearly observed in experiments focused on the growth of graphenic carbon at temperatures of 750-800^oC when the carbon precursor was introduced when the graphene growth temperature was reached. Shown in Fig. S1 is an SEM image of the pore morphology of P-Si following a carbonization step where the P-Si was heated to 800^oC prior to exposure of C_2H_2. After heating and exposure of C_2H_2 for 10 minutes at temperature, the pore morphology changes in a manner that reflects the melting of the nanostructured features of the P-Si. This is easily observed through a comparison of Fig. S1 to Fig. 1. This then confirms that the role of the temperature ramp is to catalyze carbon growth at low temperatures that can stabilize the P-Si structure for more uniform coating that occurs at higher temperatures (up to 850^oC). The use of a temperature ramp

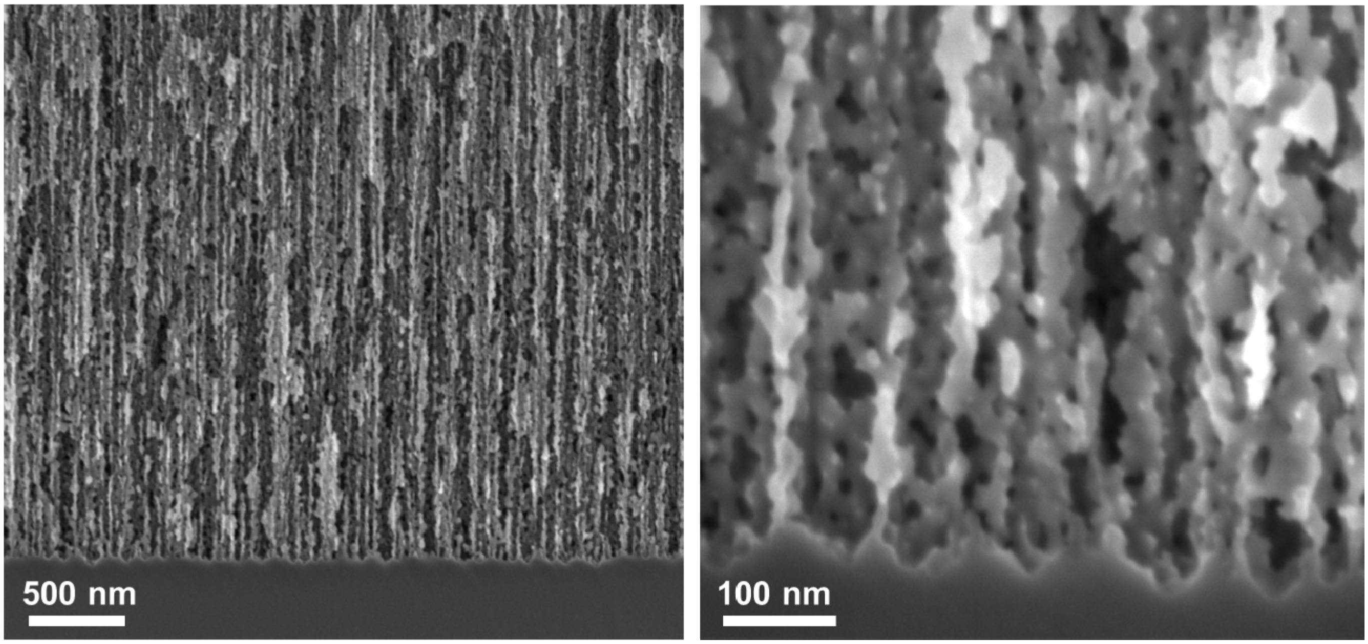

Figure S1. Cross-sectional SEM micrographs of P-Si after graphenic carbon coating at a temperature of 800°C. Note that the pore structure of the P-Si material remains in-tact, but the nanoscopic pore features indicate melting effects.

is then a critical feature to retaining surface area in the P-Si material that enables supercapacitor device fabrication. As Fig. S1 illustrates, if the surface of the P-Si was not stabilized, clear morphological changes between the uncoated and coated P-Si would be apparent in the images presented in Fig. 1.

Shown in Fig. 2 is device testing data from supercapacitor devices fabricated from P-Si after coating with graphenic carbon at a temperature of 800°C, without temperature ramping. Whereas the device performance is still significantly enhanced in comparison to uncoated P-Si, the loss of surface-area due to melting phenomena yields a decrease in the total energy density of ~ 5X compared to the P-Si coated using the same gas conditions and flows, except with a temperature ramp from 600-800°C. This demonstrates the challenge of balancing melting of the nanoscale features in P-Si that enable charge storage and the uniform, passivation of the surface to enable a conductive electrode-electrolyte interface.

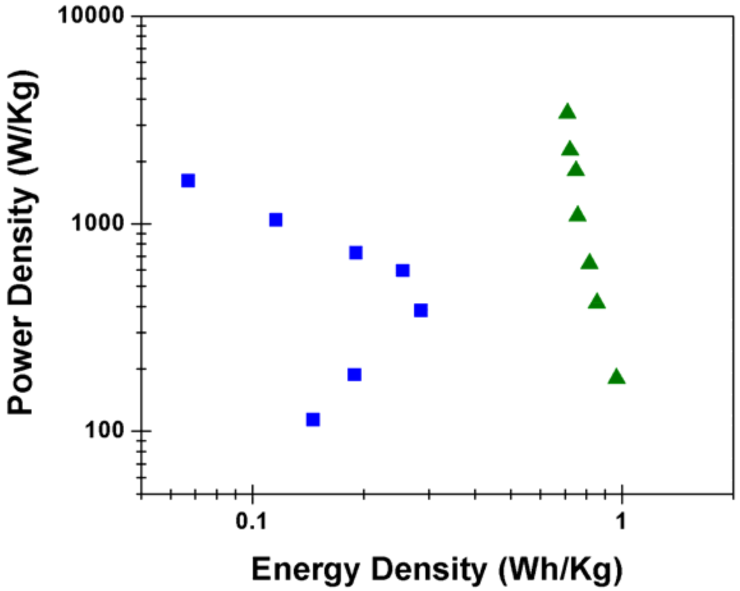

Figure S2. Ragone plot comparing specific energy and power of uncoated P-Si (blue squares) and graphenic carbon coated P-Si (green triangles) when the graphenic coating is carried out at 800°C without a temperature ramp.

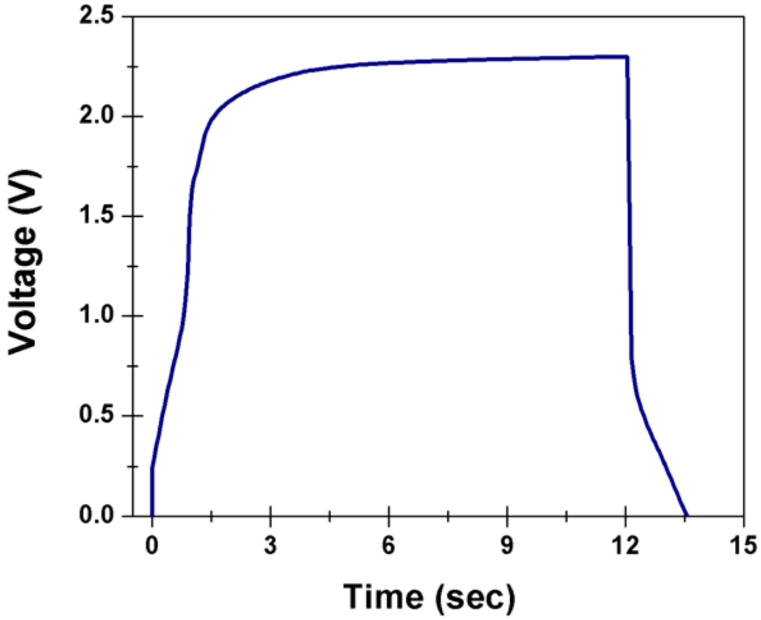

Figure S3. Galvanostatic charge-discharge cycle for pristine, uncoated P-Si charged to 2.3 V.

II. Control of graphenic carbon coating

Whereas a conformal coating applied to a material to passivate it and enable performance as a supercapacitor ideally would not change the inherent material performance in such an application, a challenge going forward for such device architectures is to preserve the surface area that is inherent in the etch process itself. This requires a unique level of control on the thickness and quality of graphene material growth that must be achieved to passivate the porous silicon structure. Whereas our coatings are found to be on average between 2-3 nm thick, (up to ~ 10 graphene layers), this notably results in the deactivation of some nanoporous regions that would play a role in double-layer energy storage if all surface area were activated for energy storage following the graphene coating. As shown in Fig. S4, TEM images emphasize some nanoporous regions that are filled with graphenic carbon, which deactivates those

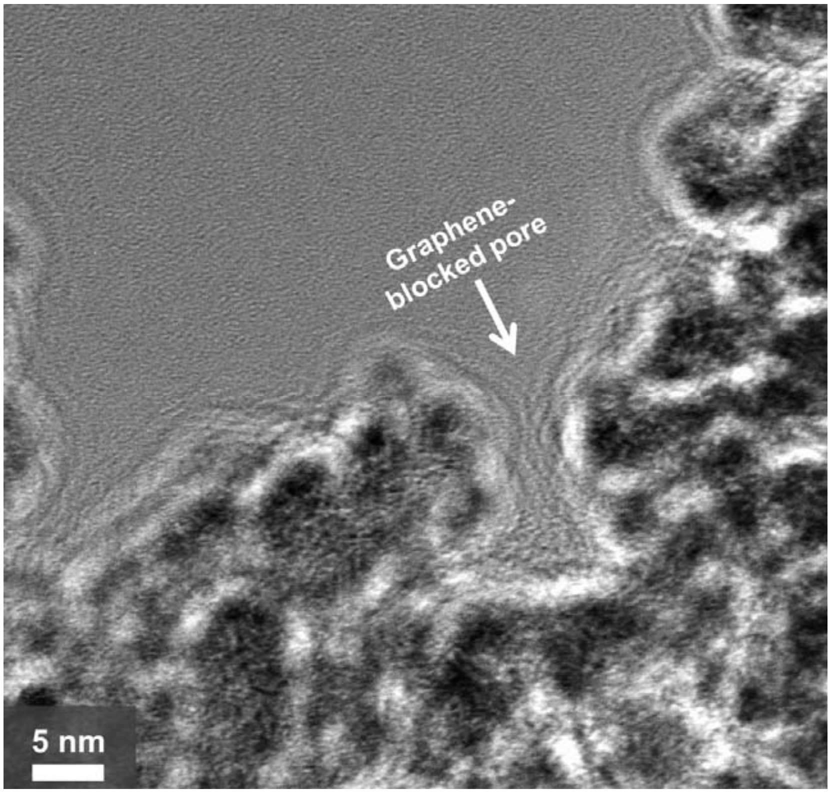

Figure S4. TEM image showing a 3-D porous silicon structure coated with graphenic carbon, where a ~ 3 nm diameter pore is being fully blocked by the coating of graphene.

pores for double-layer energy storage. The challenge going forward in optimizing these devices can be attributed both to optimizing the structure of porous silicon, as well as achieving better control on the graphene layer thickness and quality that will enable more effective passivation and the best energy storage characteristics.

III. Equivalent Circuit Modeling Results

In order to understand the results from the electrochemical impedance spectroscopic investigation presented in Fig. 2, we performed equivalent circuit analysis using Metrohm equivalent circuit analysis software. The equivalent circuit that we utilized for our modeling was a modified Randles circuit that has been widely utilized to represent electrochemical double-layer capacitors, and is shown in Fig. S5. Values obtained from the best fit of this equivalent circuit analysis are shown in Table S1.

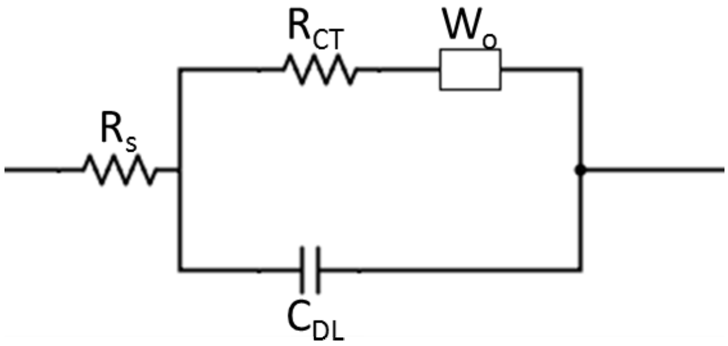

Figure S5. Equivalent circuit utilized to fit electrochemical impedance data taken for pristine and graphene coated porous silicon supercapacitors. In this model, R_s is the series resistance, R_{ct} is the charge-transfer resistance at the electrode-electrolyte interface, C_{DL} is the double layer capacitance, and W_o is the constant Warburg diffusion element. Values obtained for pristine and graphene-coated P-Si are shown Table S1.

	Pristine P-Si Device	Graphene-coated P-Si Device
**R_s (Ω)**	**233.4**	**5.2**
**C_{DL} (nF)**	**27.9**	**481.0**
**R_{CT} (Ω)**	**1596**	**49.9**
**W_o (mS)**	**0.095**	**1.45**

Table S1. Best-fit parameters to the Randles equivalent circuit model shown in Figure S5 for both pristine P-Si devices and graphene-coated P-Si devices.

The results of the equivalent circuit modeling emphasize a few key concepts that are central to a comparison between a surface-passivated and uncoated porous silicon material. First of all, the internal series resistance of the surface-passivated device decreases by almost 50X, in good agreement with through-plane electrical measurements discussed in Fig. 2. Therefore, we attribute this to the higher resistance of the etched porous silicon material, where surface defects play a role to inhibit surface conduction – a factor that is reversed when surface passivation occurs. Furthermore, we observe a ~ 20X increase in the double layer capacitance between the coated and uncoated samples, consistent with trends observed in Galvanostatic charge-discharge and cyclic voltammetry measurements. Additionally, we observe that the charge-transfer resistance, R_{ct}, is ~ 30X greater for the unpassivated, pristine porous silicon device. This resistance is simulated as being in parallel to the double-layer capacitance, and corresponds to the resistance that exists at the interface between the electrode and the electrolyte. A lower Rct value corresponds to a structure with more available sites for ion adsorption in the double layer and subsequently a lower ionic resistance at the electrode-electrolyte interface. As noted in the main text, this lower ionic resistance at the silicon-electrolyte interface is the origin of the much larger semicircle

observed in the Nyquist spectra in Fig. 2a for pristine, uncoated P-Si. Finally, the Warburg impedance element (Wo) corresponds to the ionic behavior at frequencies extending from the knee frequency (labeled in Fig. 2a) down to lower frequencies and represents a spike in the Nyquist spectra. This feature physically corresponds to the diffusion of electrolyte ions into the bulk structure of the electrode. The significantly higher W_o (where W_o has units of conductivity, in Siemens (S)) for the passivated P-Si corresponds to the more efficient diffusion of electrolyte ions into the porous structure to enable charge storage, as opposed to the uncoated, pristine P-Si devices where the value of W_o is ~ 15X smaller. Overall, this presents a picture of a comparison between two materials with identical porous structure, thickness, and materials – except one is passivated with an atomically thin layer of graphene, and this passivation can significantly improve (by over 10X) the diffusion, the electrode-electrolyte resistance, the double-layer capacitance, and the series resistance. This emphasizes surface engineering of charge storage devices as an important tool in the future of optimized device design.

IV. Cyclic Voltammetry at Different Scan Rates

In order to assess how the electrochemical window and device performance varies at different scan rates, cyclic voltammetry was carried out at 25, 50, and 100 mV/sec. These curves are shown in Fig. S6. In general, Fig. S6 emphasizes a similar trend that is evident from Galvanostatic charge-discharge curves (Fig. 3), as greater scan rates lead to less overall charge storage as evidenced by the smaller spacing between the positive and negative scan directions in the CV. The electrochemical window is also observed to not show a significant dependence on the scan rate in both pristine and graphene-coated P-Si devices.

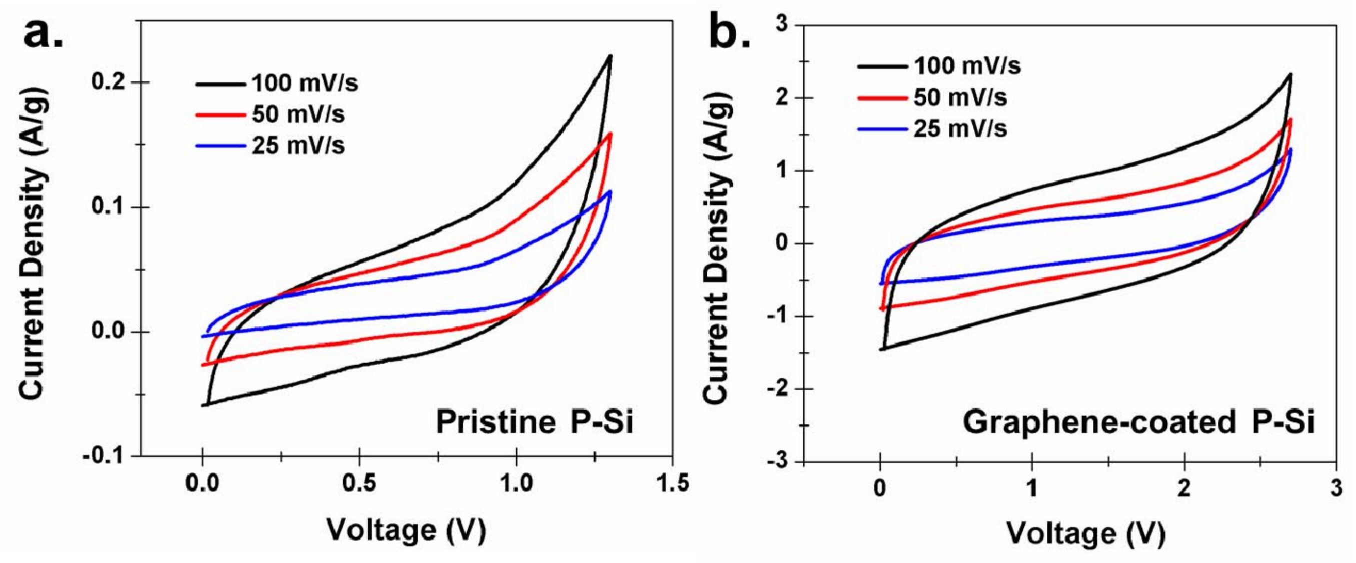

Figure S6. Cyclic voltammetry measurements taken for a. pristine and b. graphene-coated porous silicon at three voltage scan rates of 25, 50, and 100 mV/second.

V. Pristine P-Si Device Testing, 1 V

In this study, the electrochemical testing of passivated and unpassivated porous silicon devices presents a challenge in that the pristine and passivated devices exhibit different electrochemical windows for non-Faradaic energy storage. In order to present a true comparison between two otherwise identical devices, we have performed testing within the electrochemical window of the passivated device. To compliment this, we have further provided analysis of the device behavior of the unpassivated, pristine device that is performed within the electrochemical window of the pristine device, between a range of 0-1 V. This analysis is presented in Figure S7, and includes both a Galvanostatic charge-discharge measurement at different charging currents and a Ragone plot analysis of this device performance. Overall, we observe that the device performance in terms of total

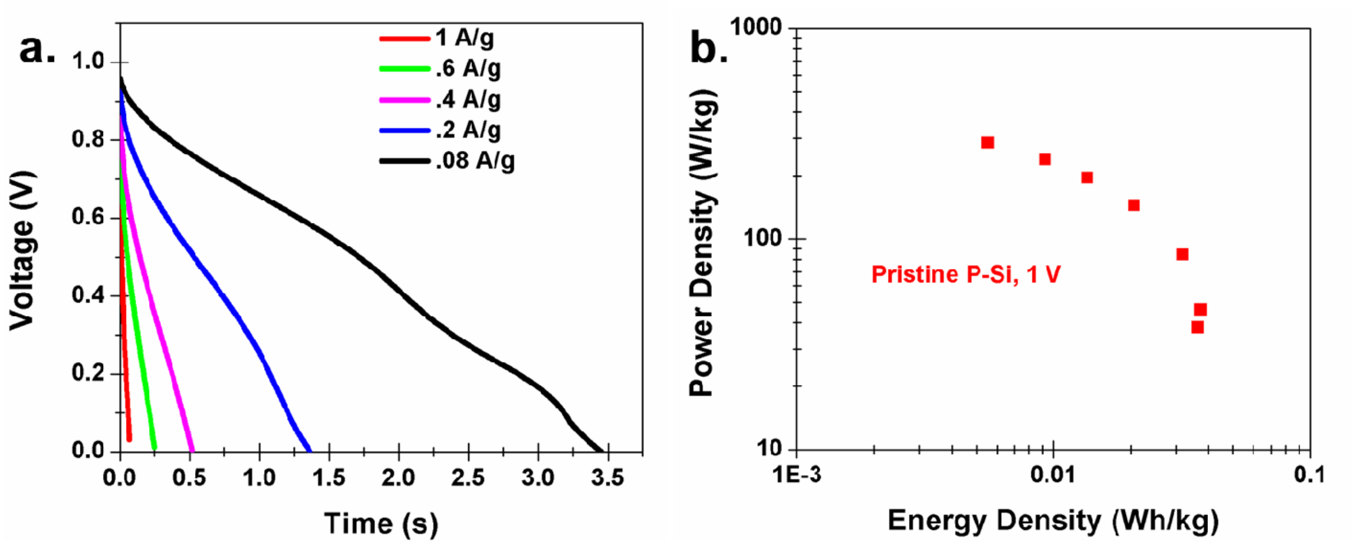

Figure S7. Device testing for pristine porous silicon devices cycled within the electrochemical window to a voltage of 1 V. a. Galvanostatic charge-discharge testing data from the pristine P-Si devices, and b. Ragone analysis of the pristine P-Si device performance.

capacitance, energy, and power capability is not significantly influenced by cycling of the device to 2.3 V. The only notable feature is the near-leveling off of the energy density of the pristine P-Si device at small charging currents in contrast to the decrease in performance in Fig. 4 that is attributed to device degradation. Additionally, the charge-discharge curves show a similar energy storage characteristic to the charge-discharge curves presented in Fig. 3, except without the presence of a large IR drop that arises due to the inability of the device to storage charge outside the electrochemical window. However, overall the performance of the device inside and outside the electrochemical window is not significantly different, only that the device performance rapidly degrades when cycled outside of the electrochemical stability window as illustrated in Fig. 3d.

VI. Porous Silicon Thickness Measurements

In order to assess the thickness of the sample, which was utilized to extrapolate the total active mass of the silicon based upon the measured porosity, we utilized SEM images to deduct the total thickness of

the porous silicon layer. Shown in Figure S8 is a side-view SEM image of the porous silicon layer produced after etching with the conditions utilized in this work. Based on analysis of this image (original scale-bars are included), the total height of the porous silicon is 4.0 microns, which is the value utilized in mass measurements.

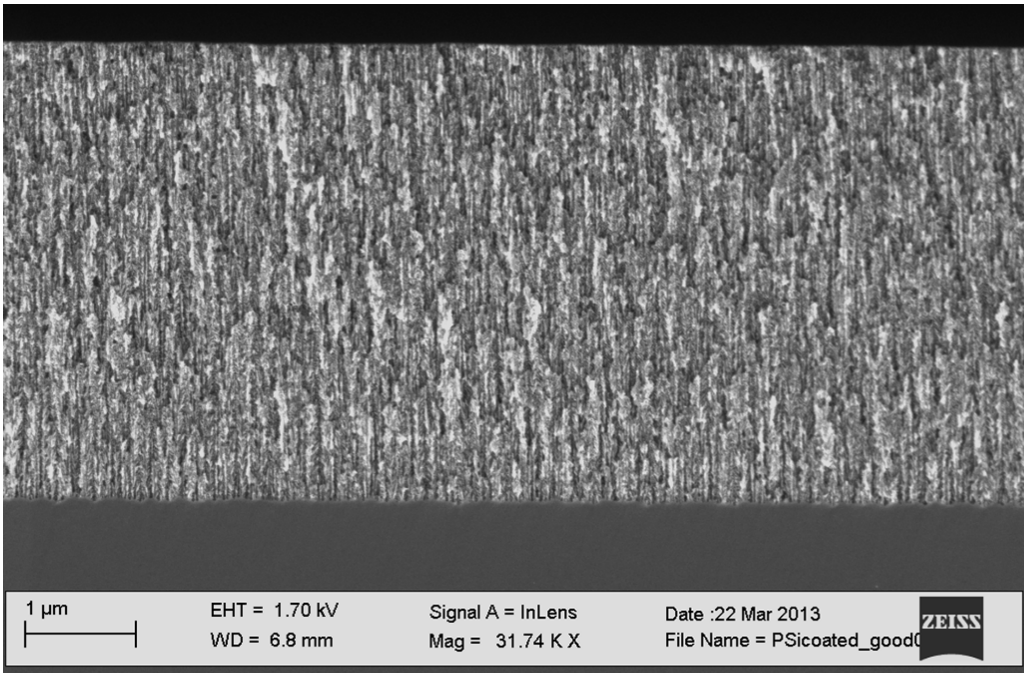

Figure S8. Side-view SEM image showing a porous silicon material etched into a silicon wafer (with original SEM scale bars) demonstrating a layer thickness of 4.0 microns.
